# Quantitative phase imaging of erythrocytes under microfluidic constriction in a high refractive index medium reveals water content changes

**DOI:** 10.1038/s41378-019-0113-y

**Published:** 2019-12-02

**Authors:** Han Sang Park, Will J. Eldridge, Wen-Hsuan Yang, Michael Crose, Silvia Ceballos, John D. Roback, Jen-Tsan Ashley Chi, Adam Wax

**Affiliations:** 1 Department of Biomedical Engineering, Duke University, Durham, NC 27708 USA; 2 Department of Molecular Genetics and Microbiology, Duke University, Durham, NC 27708 USA; 3 Duke Center for Genomic and Computational Biology, Duke University, Durham, NC 27708 USA; 4 Department of Biochemistry, Duke University, Durham, NC 27708 USA; 5 Department of Pathology and Laboratory Medicine, Emory University School of Medicine, Atlanta, GA 30322 USA

**Keywords:** Other photonics, Engineering

## Abstract

Changes in the deformability of red blood cells can reveal a range of pathologies. For example, cells which have been stored for transfusion are known to exhibit progressively impaired deformability. Thus, this aspect of red blood cells has been characterized previously using a range of techniques. In this paper, we show a novel approach for examining the biophysical response of the cells with quantitative phase imaging. Specifically, optical volume changes are observed as the cells transit restrictive channels of a microfluidic chip in a high refractive index medium. The optical volume changes indicate an increase of cell’s internal density, ostensibly due to water displacement. Here, we characterize these changes over time for red blood cells from two subjects. By storage day 29, a significant decrease in the magnitude of optical volume change in response to mechanical stress was witnessed. The exchange of water with the environment due to mechanical stress is seen to modulate with storage time, suggesting a potential means for studying cell storage.

## Sensors: Measuring blood cell deformability

A team at Duke University in the United States led by Han Sang Park has developed a novel approach to investigate the deformability of cells. The technique uses quantitative phase microscopy to measure optical volume as cells are squeezed through a constriction. The team used this approach to study changes in red blood cells that had been stored for up to 29 days. The older cells had a larger volume before constriction, and their volume changed less when they were squeezed. The increased optical volume may be due to changes in hemoglobin concentration during storage, while the smaller change in volume may be because of less water exchange during constriction of older cells. Further investigation will reveal how these biophysical properties change during storage, affecting how readily cells can pass through narrow blood vessels.

## Introduction

The flexibility and ability to endure mechanical stress are distinguishing characteristics of red blood cells (RBC) that contribute to their functionality as a transportation mechanism of gases between lungs and tissues^1^. In particular, RBCs stored for transfusion can exhibit impaired function, collectively referred to as the RBC “storage lesion”^2^, which includes alterations in rheological properties and physiological changes^3,4^. Indeed, impaired deformability of stored RBCs is a potentially serious result that affects their ability to navigate through microcirculation^5–7^. Therefore, various techniques have been used to characterize the changes in RBC’s rheological properties throughout the storage period^8–11^. Using atomic force microscopy, previous studies have shown a significant increase in Young’s modulus of the cells over the storage time indicating a decrease in the elasticity of the cells^8,9^. In addition, optical tweezers have been used by Czerwinska et al. to stretch RBCs and measure a significant increase in shear modulus over storage time, demonstrating a stiffer membrane^10^. A study by Card et al. used an ektacytometer to measure a general decrease in deformability of blood during storage by monitoring shape changes using diffractive imaging and a concentric cylinder viscometer^11^.

Another compelling approach for assessing RBC mechanical properties during storage is the use of microfluidic devices to quantify dynamic deformation of the cells^12,13^. One experiment used microfluidics with multiplexed fluidic plunger mechanisms to apply identical forces across the cells in a parallel array of funnel-shaped constrictions^12^. The deformability of individual RBCs is measured based on the pressure required to push each cell through the micrometer scale constriction. Among the different samples examined, there was significant variability in both the initial RBC deformability and storage capacity that were nonetheless accompanied by consistent storage-based deformability reduction. Finally, a recent study measured the stiffness of RBCs by imaging their deformation in the folding mode as they were flowing through microchannels^13^. Their results showed significant increases in stiffness over storage with accelerated stiffness degradation in the last 3 weeks relative to the first 3 weeks.

Here, we propose an alternative method to assess the changes in RBCs associated with storage lesion by flowing them through constricted microfluidic channels and observing nanoscale deformation with quantitative phase microscopy (QPM). QPM is a holographic method of cell imaging with nanometer path length sensitivity and the ability to observe sub-millisecond time scales^14^. Due to their homogeneous structure and uniform refractive index, RBCs have been a natural target for numerous studies using QPM^15–19^. One recent study used tomographic QPM to study changes in refractive index maps of RBCs infected with malaria parasites^15^. Studies from our group have used QPM to classify malaria infection with high accuracy using machine-learning algorithms^16^ and to quantify hemoglobin consumption by the parasite using spectroscopic information^17^. Another utility of QPM for RBC studies is to measure time series and observe membrane fluctuations. Our group used this approach to show reduced fluctuations of sickle cells relative to healthy RBCs^18^. Similarly, another independent study showed progressive reduction in temporal membrane fluctuations of stored blood over a 6-week period indicating an increase in stiffness^19^. QPM has been previously applied to studies involving microfluidic devices as well. Recent studies associated with microfluidic mixing^20^ and sperm selection^21^ show that these microchips can be integrated into a QPM system to quantify dynamic changes. Additionally, another group of researchers utilized the random rolling motions of the cells in microfluidic channels to obtain tomographic projections without mechanical scanning^22^.

In this paper, we present a quantitative analysis method for examining the biophysical response of RBCs as they are subjected to mechanical stress by flowing through constricted channels of custom-fabricated microfluidic chips and imaging these cells with QPM. Specifically, QPM is used to measure optical volume (OV) changes as the cells squeeze through the narrow channels. Unlike previous methods that have studied the rheological properties of the cells, we are able to observe efflux of water through the membrane of the cells under mechanical stress by using a high refractive index medium with QPM. Then, to illustrate the potential utility of this platform, the OV changes in response to mechanical force are compared for different storage periods to evaluate the changes in response to deformation that occur over the storage period.

## Materials and methods

### Blood collection and preparation

Following approval by the Institutional Review Board at Emory University, informed consent was obtained from two healthy donors. As previously described^23^, one unit of whole blood (500 mL ± 10%) was collected from each donor into CPD-ADSOL [AS-1] collection sets (Fenwal Lnc. Cat no. 4R3329E) and leukoreduced by an Integral SEPACELL™ RS-2000 filter. The filtered units of blood were then centrifuged at 3400 rpm for 20 min and the plasma was replaced with ADSOL [AS-1] additive solution. These processed packed RBC units, which have 99.99+% of WBCs and ~80% of plasma removed, were stored at 1–6 °C for up to 42 days. Removal of aliquots of stored RBC was accomplished anaerobically via a sterile-docking device fitted with a valve to ensure there was no re-entry of air, or other contamination, into the RBC unit. Then, the packed RBC was diluted to 0.02% hematocrit solution in 5 mL of a high refractive index medium composed of 20% bovine serum albumin (Sigma-Aldrich) in DPBS+/+ (Gibco). The refractive index of the medium was measured to be 1.372 at 23 °C using a commercial refractometer (Bellingham + Stanley). The diluted solution was then loaded on a syringe pump system (New Era syringe pump) operating at 1.5 µL/min for the squeeze experiment.

### Development of microfluidic channels

**Fig. 1:**
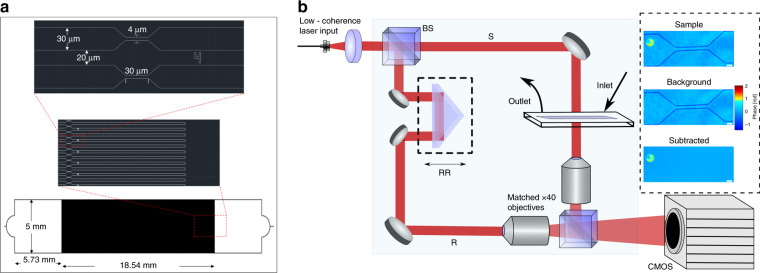
System setup. **a** Schematics of pattern mask used for photolithography. **b** Quantitative phase microscopy system: beam splitter (BS), retroreflector (RR). Path-matched sample (S) and reference (R) beams create off-axis interferograms imaged by a CMOS camera. Inset: Phase images of the sample, the background, and the RBC after background subtraction. Scale bar = 5 μm.

Glass coverslips (VWR #1.5, 22 × 40 mm) are cleaned and dried before applying SU-8 coating. First, the substrates are exposed to a piranha cleaning process for 10 min followed by rinsing with acetone and isopropanol. Then, the coverslips are dehydrated at 180 °C for 20 min on top of a hot plate. In order to achieve a 3 µm-thick coating, the coverslips are spin coated with SU8–2 (Microchem) in a headway spin coater using the following steps: SU-8 is poured on the clean substrate and distributed evenly across by spinning at 500 rpm for 5 s, followed by a spinning cycle at 1030 rpm for 45 s. For pre-soft bake, the exposed substrates are heated on top of a hotplate at 65 °C for 1 min, 95 °C for 3 min, and back to 65 °C for 1 min. The exposure is performed on a Karl Suss MA6 mask aligner using a UV lamp with an exposure intensity of 84 mJ/cm^2^. The pattern mask used for the exposure is shown in Fig. 1a. The post exposure bake is 65 °C for 1 min followed by 95 °C for 1 min and back to 65 °C for 1 min on top of a hotplate. The resist is developed in SU-8 developer for 1 min, then rinsed with isopropanol and blow-dried with N_2_. Using a profilometer (Bruker Dektak 150), the height of the developed photoresists is measured to be 3.222 + 0.109 µm. In order to enable mechanical stress to be applied to RBCs, the developed pattern has 100 openings that lead to six constriction areas each with 4 µm width. The fabricated channels are covered with glass slides (VWR, 25 × 75 × 1 mm) with both inlets and outlets created using a drill press with a diamond bit. In order to bond the cover glass to the SU-8 channels, a chamber with a customized manual press using bolted joint design^24^ is used to apply controlled pressure (2.11 MPa) with a torque wrench. While the microfluidic device is under pressure, it is heated in a convection oven at 180 °C for 1 h. Finally, blocks of polydimethylsiloxane with punched holes are bonded to the inlet and outlet of the devices using a plasma asher (Trion Technology Phantom II) to hold the tubing.

### Quantitative phase microscopy

Our custom quantitative phase microscopy system (QPM, Fig. 1b)^25^ is used to image the RBCs flowing through the microchannels. Using a customized spectral filter, broadband light from a supercontinuum laser source (Fianium SC-400-4) is filtered to a 1.12 nm full-width at half-maximum spectral bandwidth with a center wavelength at 680 nm (coherence length, *l*_c_: 204 µm).

Light at the selected wavelength is coupled into a single-mode fiber and transmitted to the off-axis Mach–Zehnder interferometer as a collimated beam. In the interferometer, the illumination light is separated by a beam splitter into sample and reference arms which are then path-matched to within the coherence length of the filtered light using mirror-based retroreflectors on translation stages. Matched microscope objectives (Zeiss Plan-NeoFLUAR ×40 0.75NA) are used in each arm, creating an image of the sample with an effective magnification of ~x107. The CMOS camera (Photron FastCam SA-4, 1024 × 1024 px, 10-bit data capture) detects the off-axis interferogram created by the angle difference between the reference and the sample arms.

In order to produce quantitative phase images, Δ*ϕ*(*x*, *y*), the interferograms are digitally processed as described in previous works^16,26^. The acquired interferograms are Fourier transformed to isolate one of the complex conjugate components by spatially filtering around the carrier frequency. Then, the filtered spatial frequency information is demodulated and inverse Fourier transformed to produce an image of the complex field containing both amplitude and phase information. Finally, the phase image is obtained by taking the angle of the complex field at each spatial location.

Since QPM obtains the complex optical field (amplitude and phase), the image can be digitally propagated to ensure they are in focus. This is an important aspect since incorrect focus can produce erroneous analysis^27^. After subtracting background noise, using a background field of view (FOV) image without any cells, the images of flowing RBCs (inset of Fig. 1b) are digitally refocused to an optimal distance as determined using the minimum variance of amplitude of the RBC for the first three frames. Then, to ensure the correct focal plane has been found, the area of the cell is calculated for each frame during its flow through the constricted channel over a range of propagation distances to determine its minimum value. The offset propagation distance found by determining the minimum area from the distance determined using minimum variance of amplitude is in agreement with results from our previous paper that examined digital focusing during stress-free flow through a channel^28^. After subtracting the background FOV that is corrected for defocus using the same determined propagation distance, any remaining phase noise only varies slowly across the field of view, arising from the temporal drift between the two interferometer arms. It is removed by fitting the background, excluding the RBCs, to third-order polynomials and subtracting. Then, changes in optical path length (OPL) of RBCs are calculated as

1 $${\mathrm{\Delta }}{\mathrm {OPL}}\left( {x,y,t} \right) = {\mathrm{\Delta }}n\left( {x,y,t} \right) \cdot h\left( {x,y,t} \right) = {\mathrm{\Delta }}\phi \left( {x,y,t} \right) \cdot \lambda /2\pi$$

where $h\left( {x,y,t} \right)$ is the height map of the object and ${\mathrm{\Delta }}n\left( {x,y,t} \right)$ is the refractive index map. The red blood cells are automatically segmented from each FOV by applying both optical path length and area thresholds from previous literature^29^. Also, in order to measure the volume changes to the RBC as it transits through the constricted channels, the ${\mathrm{\Delta }}{\mathrm {OPL}}$ is integrated to obtain optical volume (OV^27^):

2 $${\mathrm{OV}}\left( t \right) = \mathop {\iint}\limits_{x,y} {{\mathrm{\Delta }}{\mathrm {OPL}}\left( {x,y,t} \right){\mathrm {d}}x{\mathrm {d}}y} = \mathop {\iint}\limits_{x,y} {{\mathrm{\Delta }}n\left( {x,y,t} \right) \cdot h\left( {x,y,t} \right){\mathrm {d}}x{\mathrm {d}}y}$$

In our previous studies evaluating OV, we showed that this parameter was invariant for RBCs as they change orientation^28,30^. These studies examined cells over periods of 10 s or longer and showed that OV did not change by more than 2% over this period.

**Fig. 2:**
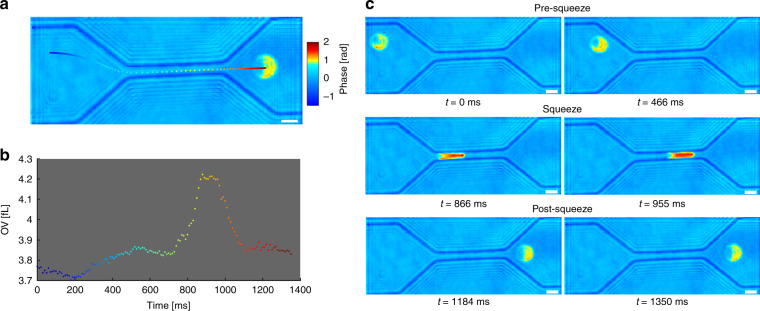
Transit through a microfluidic channel. **a** An RBC flowing through a channel while its position throughout the flow is color-coded to correspond to the temporal OV change shown in **b**. **c** Three different segments (pre-squeeze, squeeze, and post-squeeze) of the transit through the constricting channel. Scale bar = 5 μm (see Mov 1 for video).

An example of the transit through the constricted channel as well as corresponding OV measurements throughout the flow are shown in Fig. 2a, b. In order to quantify volume changes during the flow, total transit time is manually divided into three different segments as pre-squeeze, squeeze, and post-squeeze as shown in Fig. 2c. The three segments are selected by ensuring that the cells are not mechanically stressed nor in contact with the walls during the pre-sqeeze and post-squeeze segment and making sure that the entire cell is within the constricting channel during the squeezing segment.

## Results

In order to observe the biophysical changes to RBCs over the storage period, two different blood samples are imaged at three time points: day 1, 15, and 29. The number of cells imaged on each day for the two samples are shown in Table 1.

**Table 1:** Sample size.

	Day 01	Day 15	Day 29
Sample 01 (Female, age: 69)	66	95	94
Sample 02 (Male, age: 51)	96	42	79

**Fig. 3:**
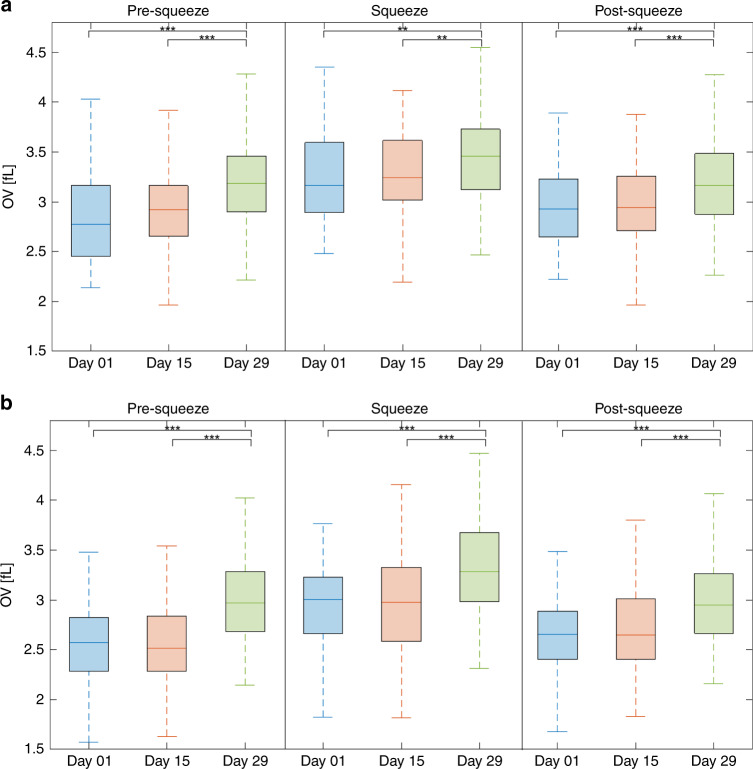
Box plots of RBC’s OV flowing through the microchannels. **a** Sample 01 and **b** Sample 02 (** for *P* < 0.01; *** for *P* < 0.001).

The boxplots of OV for the three different mechanical stress states at all the storage time points for the two samples are shown in Fig. 3a, b

As can be seen in Fig. 3, the OV of the RBCs between day 1 and day 15 does not show statistically significant difference throughout the three stress states for both samples. In contrast, the differences in OV between day 1 and day 29, as well as between day 15 and day 29, are statistically significant for both samples. These results arise from a general increase in OV at day 29 relative to the other earlier storage time points, regardless of the mechanical stress state. This trend can be seen in Table S1 in the supplementary information with the average as well as the standard deviation of the populations in Fig. 3.

**Fig. 4:**
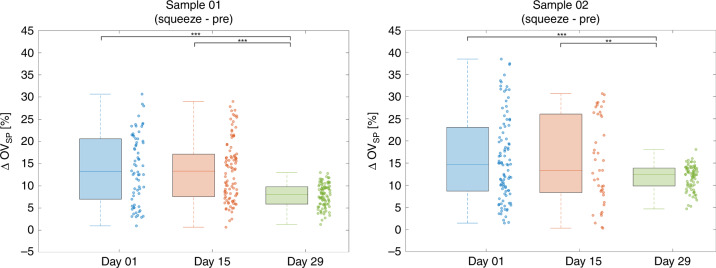
Box plots and scatter plots of ∆OV_SP_.

An increase in OV is observed during the squeeze state relative to both pre-squeeze and post-squeeze state regardless of the storage day. In order to further analyze this trend at the single-cell level, the biomechanical properties of RBCs during storage are further quantified by calculating the percentage change in OV during the squeeze segment relative to the OV before entering the constricted channel (Fig. 4). The percentage change in OV between the two states, ΔOV_SP_, is calculated as

3 $$\Delta {\mathrm {OV}}_{{\mathrm {SP}}} = \frac{{\left( {\overline {{\mathrm {OV}}} _{{\mathrm {Squeeze}}} - \overline {{\mathrm {OV}}} _{{\mathrm {Pre}}}} \right)}}{{\overline {\mathrm {{OV}}} _{\mathrm {{Pre}}}}} \times 100$$

where $\overline {\mathrm {{OV}}} _{\mathrm {{Squeeze}}}$ and $\overline {\mathrm {{OV}}} _{\mathrm {{Pre}}}$ are the average OV of the flow segments during and before the constricted channel, respectively.

Similar to the OV comparison in Fig. 3, there is no significant difference for ΔOV_SP_ between day 1 and day 15. In contrast, there are statistically significant differences in ΔOV_SP_ between day 1 and day 29 as well as between day 15 and day 29. No correlation was observed between the ΔOV_SP_ for a given cell and its original optical volume (Supplementary Information). The average ΔOV_SP_ as well as the corresponding standard deviation and the observed range in values for the data in Fig. 4 are shown in Table S2 in the supplementary information.

As can be seen in Fig. 4, the positive ΔOV_SP_ values indicate that there is an increase in OV as the cells are squeezed through the constricted channel. Also, it should be noted from the measured data above that the ΔOV_SP_ values, as well as the corresponding standard deviation and ranges, are much larger at day 1 and 15 than those at day 29 for both samples. The decrease in the variability of ΔOV_SP_ over storage time could be an important storage-based characteristic resulting from a population distribution shift from a mixture of younger and older cells to a population composed mostly of the older RBCs.

**Fig. 5:**
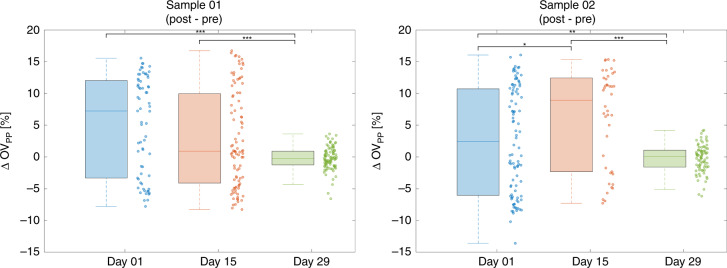
Box plots and scatter plots of ∆OV_PP_.

Further insight can be gained by examining the percentage change in OV after leaving the constriction channel compared to the OV before entering the channel (Fig. 5). The percentage change in OV these two states, ΔOV_PP_, is calculated as

4 $$\Delta {\mathrm {OV}}_{{\mathrm {PP}}} = \frac{{\left( {\overline {\mathrm {{OV}}} _{\mathrm {{Post}}} - \overline {\mathrm {{OV}}} _{\mathrm {{Pre}}}} \right)}}{{\overline {\mathrm {{OV}}} _{\mathrm {{Pre}}}}} \times 100$$

where $\overline {\mathrm {{OV}}} _{\mathrm {{Post}}}$ is the average OV of the flow segment after the constricted channel.

Except for the difference in ΔOV_PP_ between day 1 and 15 for sample 1, there are significant differences between the other storage times. No correlation was observed between the ΔOV_PP_ for a given cell and its initial OV (Supplementary Information). The average as well as the corresponding standard deviation and the range of the populations in Fig. 5 are shown in Table S3 in the supplementary information.

The ranges of ΔOV_PP_ at day 1 and 15 are larger than the ΔOV_PP_ at day 29, as shown in Fig. 5, resembling the trend shown in Fig. 4 for ΔOV_SP_. It should be also noted that the average ΔOV_PP_ is closer to 0 at day 29 than for the other time points indicating that more cells are able to recover the original OV after squeezing through the channels.

## Discussion

Numerous studies have examined storage-based changes in rheological properties of RBCs and have shown decreased deformability in RBCs associated with oxidative stress^31^ and increased mean corpuscular hemoglobin concentration^32^. In addition, the recent study by Alshalani et al. emphasized the importance of monitoring water permeability as a potential predictor to assess the quality of the RBCs during hypothermic storage^33^. Using QPM, we analyzed RBCs flowing through constrictive microfluidic channels after different lengths of storage time. Here, we observed changes in optical volume, which can be an indication of transport of water from the cells. This unique observation was enabled by using a high refractive index medium and a fixed channel height. Thus, when the cells were mechanically compressed, the density was seen to increase relative to the background as water was displaced. This platform could further our knowledge of the deformability of RBCs, their ability to transfer fluids with their environment and the impact of constriction during circulation through small microvasculature.

In this paper, we have examined how RBCs respond to mechanical stress. With further development, this platform could be applied to the study of stored blood units. The current Food and Drug Administration guideline for 42-day RBC storage interval is based on recovery of over 75% of transfused RBCs in circulation at a time point of 24 h post-transfusion^2^. The benchmark assumes that RBCs which remain in circulation after the 24-h window will have normal lifespans^34^. However, their presence in circulation alone does not necessarily indicate their ability to function as a transportation vehicle of oxygen to tissue. Since the majority of oxygen exchange occurs in the microvasculature, it is important to assess the ability of RBCs to tolerate high pressure while flowing through narrow capillaries much smaller than their own diameters^1^. We believe that measurement of OV changes for RBCs under mechanical forces as a function of storage time could be applicable to assessment of stored cells.

As can be seen in Fig. 3, there are statistically significant differences between the OV of the cells at day 1 and 29 as well as between those at day 15 and 29. The differences between the populations are due to an increase in OV of the cells at day 29 relative to those at day 1 and 15. The increase in OV can be correlated to findings of a previous study by Park et al.^35^, where an increase in hemoglobin concentration was measured for stored RBCs. The study showed a significant increase in hemoglobin concentration over the storage period without a significant change in total hemoglobin content over the same period, an effect that could be accounted for by water efflux through the membrane. A similar increase in density throughout the lifecycle of RBCs in circulation has been reported in previous studies as well^36–39^.

As shown above in Eq. (2), OV is derived from both the cell’s physical height as well as its refractive index difference relative to the medium. We now consider the relative contribution of each component. By assuming uniform refractive index throughout the RBC, OV can be expressed as

5 $${\mathrm{OV}}\left( t \right) = \Delta \overline n \left( t \right) \cdot {V}\left( t \right) = \left( {\overline n _{\mathrm {{RBC}}}\left( t \right) - n_{\mathrm {m}}} \right) \cdot {V}\left( t \right)$$

where $\Delta \overline n \left( t \right)$ is the average refractive index difference of the cell and *V*(*t*) is the physical volume of the cell. Furthermore, the average refractive index difference $\Delta\overline{n}$ is derived by subtracting the refractive index of the medium used in the experiment, *n*_m_, from the average refractive index of the cell, $\bar n_{{\mathrm {RBC}}}\left( t \right)$. Therefore, the optical volume change over the storage time can be expressed as a ratio as

6 $${\mathrm {OV}}_{\mathrm {{ratio}}}\left( t \right) = \frac{{\left( {\overline n _{\mathrm {{RBC}}}\left( {t_{\mathrm {f}}} \right) - n_{\mathrm {m}}} \right)}}{{\left( {\overline n _{\mathrm {{RBC}}}\left( {t_{\mathrm {i}}} \right) - n_{\mathrm {m}}} \right)}} \cdot \frac{{{V}\left( {t_{\mathrm {f}}} \right)}}{{{V}\left( {t_{\mathrm {i}}} \right)}}$$

**Fig. 6:**
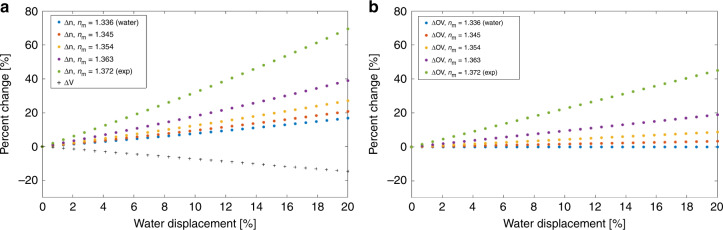
∆OV simulation. **a** Δ*n* and Δ*V* over displacement of water from RBC at different *n*_m_. **b** ΔOV over displacement of water from RBC at different *n*_m_.

As can be seen in Eq. (6), the change in OV over time can be sensitive to the change in refractive index of the cell by selecting the refractive index of the medium, *n*_m_. By using a higher *n*_m_, closer to the refractive index of the cell rather than that of water, any volume decrease due to the displacement of water from the cell can be made to have a lesser effect than the increase in the refractive index difference due to a higher concentration of hemoglobin. In order to demonstrate the expected OV change as a function of the volume of water displaced from the cell, a RBC model was simulated using typical values from the literature (given in Supplementary Information) and is studied in Fig. 6.

As can be seen in Fig. 6a, the rate at which the refractive index difference increases due to water displacement becomes more pronounced as the medium refractive index, *n*_m_ is increased compared to that of water. Also, the physical volume *V* decreases linearly due to water loss regardless of the choice of *n*_m_. Hence, as the refractive index of the selected medium *n*_m_ increases, the relative effect due to refractive index difference becomes greater than the effect of the reduction in physical volume due to water loss. This concept is reflected in the overall percent change in OV as shown in Fig. 6b. Therefore, the increase in OV over storage time in Fig. 3 is believed to be largely due to a change in refractive index, as a result of the efflux of water through the membrane, producing an effective net increase in hemoglobin concentration. The correlation between the water content change and the optical volume change was validated with the osmolarity experiment shown in the supplementary information. The results of the experiment showed that changes in water content of cell can be estimated using optical volume as measured with the system.

Similarly, the increase in OV during the squeeze segment of the flow, as shown in Figs. 3 and 4, can also be explained by displacement of water from the cells under stress. As they flow through the constricting channel, the RBCs experience mechanical pressure which forces them to discharge water. A previous study by Gambhire et al. have also suggested possible volume loss by RBCs during their transition through constricting PDMS channels, simulating RBCs passing through inter-endothelial slits, as an explanation for the transition of RBCs with high sphericity index^40^. Interestingly, cells at day 1 and day 15 show a larger range of ΔOV_SP_ relative to the cells at day 29. This can be explained by considering that over the storage period, the population of RBCs shifts from a mixture of younger and older cells towards a population distribution consisting of largely older cells. Hence, the reduction in the range of OV change, corresponding to less water discharge under stress, could be perhaps be used as an indicator of aging RBCs during storage.

Another interesting aspect of our findings is the degree to which RBCs recover their OV after stress. The ranges of ΔOV_PP_, shown in Fig. 5, are larger at day 1 and day 15 than that at day 29 as the average ΔOV_PP_ at day 29 is closer to 0. Due to a general decrease in water displacement under mechanical stress at day 29, more cells are able to recover the original OV after the transit through the constricting channels. It should be noted that a previous study by Sun et al.^13^ has shown significant increase in stiffness of RBCs over the storage time especially by week 4. Similarly, our experiment shows significant changes in ΔOV_SP_ and ΔOV_PP_ by day 29, corresponding to less exchange of water with the medium by RBCs under stress. Both of these rheological properties, which directly affect cell deformability, show significant change over storage across a similar time period. In order to show a relationship between the rheological properties, RBCs were chemically degraded through glutaraldehyde treatment and imaged with the system as shown in the supplementary information (Fig. S4). There was a significant increase in OV for those cells treated with glutaraldehyde in a dose-dependent manner, possibly due to an increase in stiffness as a result of an increase in the viscosity of the cytoplasm. Further analysis of the relationship between these parameters of the cells as well as their changes over storage could have an impact on monitoring of stored blood by distinguishing older cells that may not be as effective in transiting through microvasculature.

## Conclusion

In this paper, we measured OV changes of RBCs as they transit through constricting channels of microfluidic devices at different storage times using QPM. RBCs that had been stored for up to 29 days showed a general increase in OV corresponding to the known increase in hemoglobin concentration over the storage period. In addition, OV increased as the RBCs were squeezed in the narrow channels also due to higher hemoglobin concentration arising from water displacement through the membrane in response to mechanical stress. Interestingly, at day 29, significant reduction in OV changes relative to earlier time points were observed, possibly implying less water exchange by an RBC population composed of older cells. The change in the biophysical response of the cells over a storage period can impact their ability to effectively travel through narrow microvessels. The results shown here suggest that QPI in combination with constrictive microfluidics and high index media can be used to monitor unique aspects of RBC biomechanics with potential importance to evaluation and monitoring of stored blood.

## Supplementary information

Supplementary information

Movie 1
