# Supplementary material for: Quantitative phase imaging of erythrocytes under microfluidic constriction in a high refractive index medium reveals water content changes
Source: Microsyst Nanoeng. 2019 Dec 2;5:63. doi: 10.1038/s41378-019-0113-y (PMC6885519; doi:10.1038/s41378-019-0113-y)
Supplement: Supplementary file 1 — Supplementary information [file 41378_2019_113_MOESM1_ESM.pdf]

**Supplementary materials**

**Figure 7 simulation**

In order to demonstrate the changes in OV due to displacement of water through the membrane, typical RBC values from previous literature are used to create a simple model of the cell. The simulation does not utilize QPM data from the experiment to avoid using assumptions along with the data to derive these parameters. The simulation shows a possible increase in OV of an average RBC due to efflux of water from the cells in a high refractive index medium.

Firstly, the number of hemoglobin molecules within the RBC model is calculated using known parameters from previous articles. Using mean corpuscular hemoglobin concentration (MCHC) of a RBC, 330g/L [1], and molecular weight (MW) of hemoglobin (Hb), 64458 g/mol [2], initial molar concentration of Hb can be derived as follows,

$$[\mathrm{Hb}]_i = \frac{MCHC}{MW}$$

Then, mean corpuscular volume (MCV) of a RBC, 90e-15L [3], is used along with the concentration of Hb, $[\mathrm{Hb}]_i$ ,to calculate the number of Hb molecules as shown below,

$$\mathrm{Hb}_i = \mathrm{MCV} \cdot [\mathrm{Hb}]_i$$

Using the $\mathrm{Hb}_i$ and the molar volume (MV) of Hb, 48.227 L/mol [4], the initial volume of Hb can be calculated as

$$V_{Hb}^{i} = Hb_i \cdot MV$$

The volume fraction of Hb using the calculated $V_{Hb}^{i}$ and MCV is 0.247 which matches values found in literature, 0.25 [1]. The volume fraction of water is reported to be 0.72 [5] and can be used to calculate the initial volume of water in the RBC as

$$V_{H_2O}^{i} = MCV \cdot 0.72$$

Then, using the refractive index of water, 1.336, along with the refraction increment of Hb, α = 0.144 ml/g [6], Barer's expression can be used to estimate the initial refractive index of the RBC following,

$$n_{RBC}^{i} = n_{H_2O} + \alpha \cdot MCHC$$ [6]

Finally, using these values, changes in $n_{RBC}$ as well as OV changes due to the efflux of water from the cell can be calculated. The new RBC volume after water displacement is calculated as,

$$V_{RBC}^{f} = \mathrm{MCV} - V_{H_2O}^{i} \cdot percent\ displacement$$

Then, the new concentration of Hb of the cell can be derived by,

$$[\mathrm{Hb}]_f = \frac{\mathrm{Hb}_i}{V_{RBC}^{f} \cdot MW}$$

$[\mathrm{Hb}]_f$ is then used to obtain the new refractive index of the RBC via Barer's expression,

$$n_{RBC}^{f} = n_{H_2O} + \alpha \cdot [Hb]_f$$

Subsequently, the Δn ratio, change in refractive index ratio, as well as the physical volume ratio in Figure 7a can be found using,

$$\Delta\text{n ratio} = \frac{n_{RBC}^{f} - n_{medium}}{n_{RBC}^{i} - n_{medium}}$$

$$\text{V ratio} = \frac{V_{RBC}^{f}}{MCV}$$

Ultimately, the OV change in Figure 7b is calculated using,

$$\text{OV change} = \Delta\text{n}_{ratio} \cdot \text{V}_{ratio}$$

**Osmolarity experiment**

In order to correlate the change in optical volume to the displacement of water, stationary RBCs were imaged in 3 separate media with varying osmolarity of 200mOsm, 300mOsm, and 400mOsm. The osmolarity of the medium was controlled by diluting DPBS+/+ 1x and 10x with distilled water. Then, bovine serum albumin was mixed to the solutions and the refractive indexes were measured to be consistent at 1.373 ± 9.5e-4. Figure S1 below shows the boxplots of the OV of the RBCs in the 3 different medium.

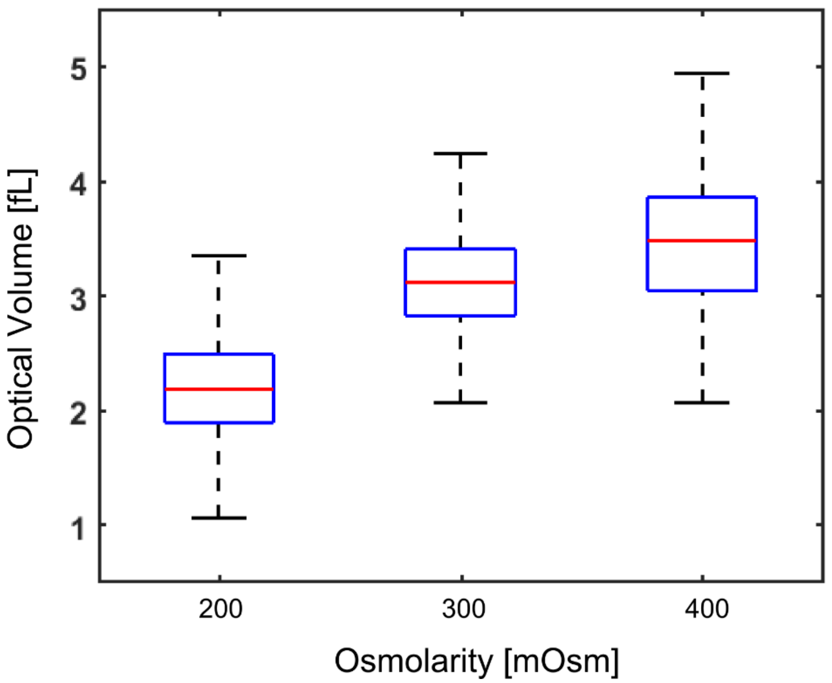


**Fig S1 Osmolarity Experiment** Optical volume of RBCs in media of varying osmolarity: 200mOsm, 300mOsm, and 400mOsm (N = 270, 175, and 182 cells respectively)

As can be seen in Fig S1, when the RBCs are in the hypotonic solution in which water enters the cells, the OV of the cells is smaller compared to that of the cells in the isotonic (300 mOsm) solution. This corresponds to a *lower* internal density of the cell since the increased size of the cell displaces the high index medium which previously occupied this volume, producing a smaller net phase change and in turn a lower OV. In contrast, when the water is displaced from the cell in the hypertonic solution, the optical volume increases relative to the cells at equilibrium. In this case, as water is displaced from the cell, it shrinks, and that volume is now occupied by high index medium, producing an increase in net phase and a higher OV.

The average OV of the cells in the hypotonic, isotonic, and hypertonic solutions are 2.22 $\pm$ 0.5fL, 3.17 $\pm$ 0.6fL, and 3.51 $\pm$ 0.7fL respectively. A 30% decrease in the OV of the cells in the hypotonic solution relative to the cells in the equilibrium, and a 10.7% increase in the OV of the cells in the hypertonic solution was found. Using the measured MCV and MCHC of the subject at 80.8fL and 36.5g/dL respectively with a hematology analyzer, the percent change in the water content relative to the optical volume, shown in Fig S2 below, can be simulated following the method described in the previous section.

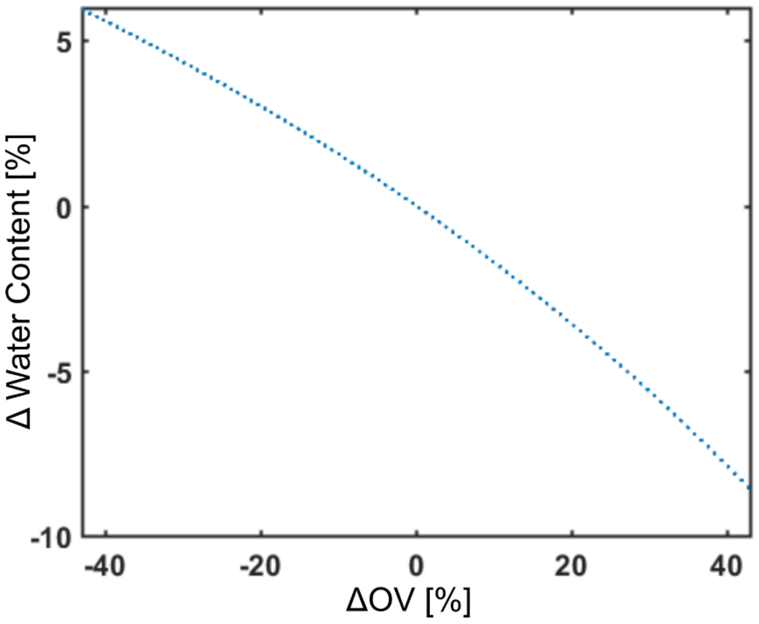


**Fig S2 ΔWater Content vs. ΔOV** Change in optical volume and corresponding change in water content of a cell

As can be seen in Fig S2, for the average 30% OV decrease as seen in our hypertonic experiments, there was an expected increase of 4.3% in the water content of the cell. For our hypotonic experiments where an average 10.7% increase in the OV was seen, there is an expectation of a 1.85% decrease in the water content of a cell. The changes in water content predicted by the simulation for the observed OV changes are comparable to water content changes with varying osmolarity in previous literature[7]. These earlier experiments measured a 10% increase in the water content in the solution with osmolarity of 200mOsm. In addition,

when fit to an exponential function, the water content decrease at 400mOsm was estimated to be 2.9%.

The discrepancy between our simulation and the water content of cells from the previous literature are likely due to the earlier work providing only a bulk estimate of a cell population. In contrast, our work is evaluating individual cells. As can be seen in Figure S1, there is a large range of OV for each set of cells at a given osmolarity. Further experiments can better help link single cell measurements with those for bulk cell populations However, these experiments help confirm that a change in the water content of the cell can be observed with optical volume measurements using quantitative phase imaging and a high refractive index medium.

### $OV_{Pre}$ vs $\Delta OV_{SP}$ / $OV_{Pre}$ vs $\Delta OV_{PP}$

In order to show that the OV change induced by mechanical stress through the constricted channel is not correlated to the increase in optical volume over storage time due to increase in hemoglobin, initial OV before squeeze is plotted against both $\Delta OV_{SP}$ and $\Delta OV_{PP}$ in the Fig S3 below.

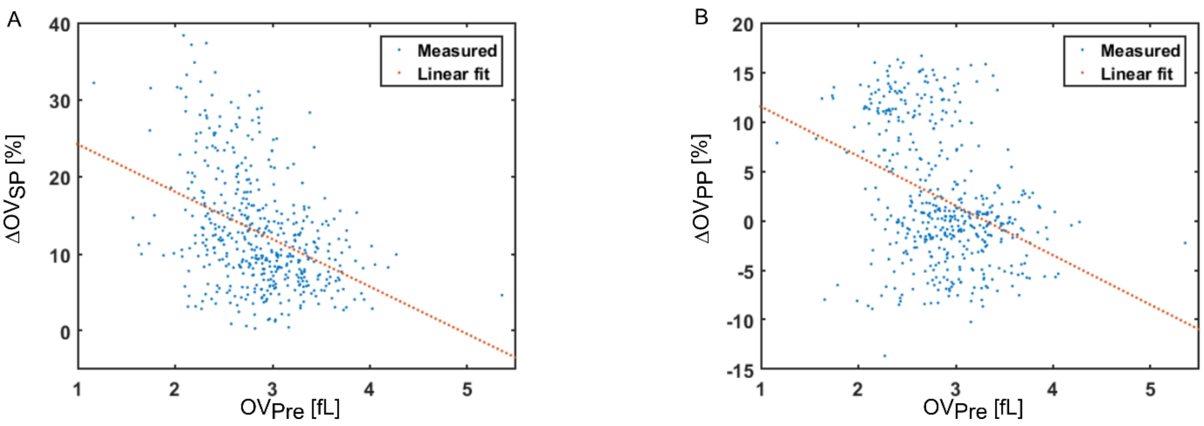


**Fig S3 A) $OV_{Pre}$ vs. $\Delta OV_{SP}$** Correlation between the initial optical volume of the cells and change in optical volume during squeeze relative to the initial volume **B) $OV_{Pre}$ vs. $\Delta OV_{PP}$** Correlation between the initial optical volume of the cells and change in optical volume post-squeeze relative to the initial volume

As can be seen, there is no correlation between $OV_{Pre}$ and $\Delta OV_{SP}$ as well as between $OV_{Pre}$ and $\Delta OV_{PP}$ where the R-squared values of the linear fits were 0.16 and 0.13 respectively. Therefore, the OV changes induced by the transit through the constricting channel are independent of the optical volume changes over the storage time.

### Chemical degradation through glutaraldehyde treatment

Glutaraldehyde treatment has been used as chemical means to degrade RBCs. Given our observations, the RBC storage and the artificial degradation lead to an increase in optical volume. In order to show the changes in the OV of the cells during chemical degradation, RBCs were treated with glutaraldehyde (0.01% and 0.05% v/v) following the protocols by Boas *et al* [8]. Then, stationary RBCs in high refractive index medium were imaged using the QPI system.

Figure S4 below shows the boxplots of the OV of the untreated RBCs as well as the RBCs treated with glutaraldehyde at different concentrations.

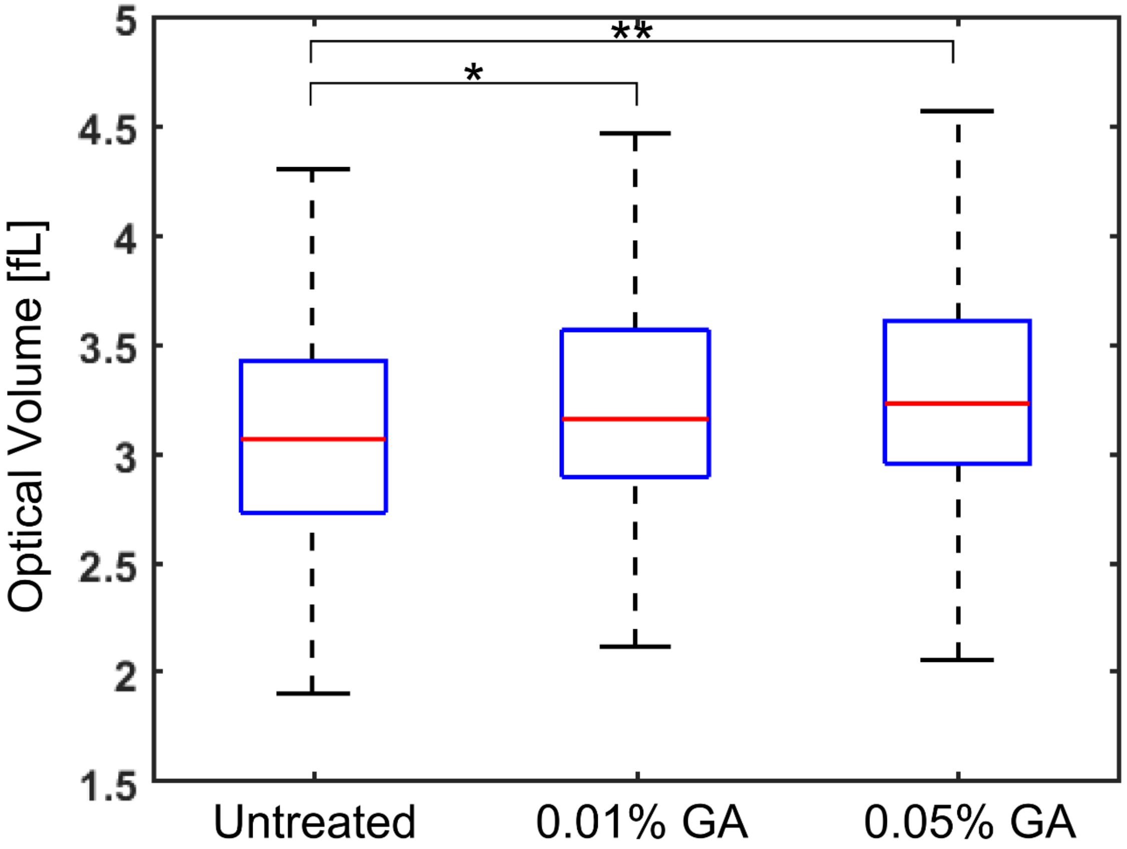


**Fig S4 Optical volume vs glutaraldehyde treatment** Optical volume bar plots of untreated RBCs and those treated with 0.01% and 0.05% glutaraldehyde (N = 201, 173, and 211 cells respectively)

As can be seen in Fig S4, there was a significant difference in OV between the untreated RBCs and those treated with 0.01% of glutaraldehyde (p-value: 0.036) as well as those treated with 0.05% of glutaraldehyde (p-value: 0.0013). The increased difference of OV shows a dose-dependence to the glutaraldehyde concentration. The significant difference in OV between the untreated cells and cells treated with glutaraldehyde may be due to an increase in the viscosity of cytoplasm of the cells as an effect of the glutaraldehyde treatment shown in previous study by Forsyth *et al* [9]. The changes in the OV of the cells through the artificial aging should be explored further in future studies to understand the rheological changes of the cells over storage time.

**Tables**

**Table S1 – Average OV and standard deviation for sample 1 and 2**

| **Sample 01** (Average ± sd [fL]) | Day 01 | Day 15 | Day 29 |
|---|---|---|---|
| Pre-squeeze | 2.84 ± 0.44 | 2.89 ± 0.44 | 3.21 ± 0.45 |
| Squeeze | 3.22 ± 0.45 | 3.27 ± 0.46 | 3.46 ± 0.48 |
| Post-squeeze | 2.97 ± 0.41 | 2.96 ± 0.41 | 3.20 ± 0.45 |

| **Sample 02** (Average ± sd [fL]) | Day 01 | Day 15 | Day 29 |
|---|---|---|---|
| Pre-squeeze | 2.57 ± 0.44 | 2.55 ± 0.41 | 2.99 ± 0.41 |
| Squeeze | 2.96 ± 0.46 | 2.94 ± 0.50 | 3.34 ± 0.48 |
| Post-squeeze | 2.62 ± 0.42 | 2.69 ± 0.40 | 2.98 ± 0.43 |

**Table S2 – Average, standard deviation, and range of $\Delta OV_{SP}$ for sample 1 and 2**

| Average ± sd [%] (Min ~ Max) | Day 01 | Day 15 | Day 29 |
|---|---|---|---|
| Sample 1 | 13.9 ± 7.7 (0.95 ~ 30.7) | 13.4 ± 7.0 (0.67 ~ 29.0) | 7.82 ± 2.6 (1.28 ~ 13.0) |
| Sample 2 | 16.0 ± 9.7 (1.48 ~ 38.4) | 15.4 ± 9.7 (0.36 ~ 30.6) | 11.8 ± 2.8 (4.68 ~ 18.1) |

**Table S3 – Average $\Delta OV_{PP}$, standard deviation as well as corresponding range for sample 1 and 2**

| Average ± sd [%] (Min ~ Max) | Day 01 | Day 15 | Day 29 |
|---|---|---|---|
| Sample 1 | 4.95 ± 7.7 (-7.76 ~ 15.6) | 2.83 ± 7.6 (-8.28 ~ 16.7) | -0.21 ± 1.8 (-6.56 ~ 3.61) |
| Sample 2 | 2.60 ± 8.2 (-13.6 ~ 16.0) | 6.20 ± 7.6 (-7.29 ~ 15.3) | -0.23 ± 2.2 (-6.16 ~ 4.19) |

**MOV 1 RBC flow through constricted channel** (top) RBC flow through the channel (bottom-left) refocused, background-subtracted, and segmented RBC synchronized to the flow at the top (bottom-right) OV change synchronized to the flow

**References**


1. Stadler, A. M. *et al.* Hemoglobin Dynamics in Red Blood Cells: Correlation to Body Temperature. *Biophys. J.* **95**, 5449–5461 (2008).
2. Van Beekvelt, M. C. P., Colier, W. N. J. M., Wevers, R. A. & Van Engelen, B. G. M. Performance of near-infrared spectroscopy in measuring local O2 consumption and blood flow in skeletal muscle. *J. Appl. Physiol.* **90**, 511–519 (2001).
3. McPherson, Richard A., and Matthew R. Pincus. *Henry's Clinical Diagnosis and Management by Laboratory Methods E-Book*. Elsevier Health Sciences, (2017).
4. Jin, X. *et al.* Erythrocyte Enrichment in Hematopoietic Progenitor Cell Cultures Based on Magnetic Susceptibility of the Hemoglobin. *PLOS ONE* **7**, e39491 (2012).
5. Levin, R. L., Cravalho, E. G. & Huggins, C. E. Effect of hydration on the water content of human erythrocytes. *Biophys. J.* **16**, 1411–1426 (1976).
6. Zhernovaya, O., Sydoruk, O., Tuchin, V. & Douplik, A. The refractive index of human hemoglobin in the visible range. *Phys. Med. Biol.* **56**, 4013 (2011).
7. Kageyama, K., Onoyama, Y., Kogawa, H., Goto, E. & Tanabe, K. The maximum and minimum water content and cell volume of human erythrocytes in vitro. *Biophys. Chem.* **34**, 79–82 (1989).
8. Boas, L. V. *et al.* Assessment of the Deformability and Velocity of Healthy and Artificially Impaired Red Blood Cells in Narrow Polydimethylsiloxane (PDMS) Microchannels. *Micromachines* **9**, 384 (2018).
9. Forsyth, A. M., Wan, J., Ristenpart, W. D. & Stone, H. A. The dynamic behavior of chemically “stiffened” red blood cells in microchannel flows. *Microvasc. Res.* **80**, 37–43 (2010).
